# Interleukin-12 (IL-12)/STAT4 Axis Is an Important Element for β-Cell Dysfunction Induced by Inflammatory Cytokines

**DOI:** 10.1371/journal.pone.0142735

**Published:** 2015-11-10

**Authors:** Jessica R. Weaver, Jerry L. Nadler, David A. Taylor-Fishwick

**Affiliations:** 1 Department of Microbiology and Molecular Cell Biology, Eastern Virginia Medical School, Norfolk, Virginia, United States of America; 2 Department of Internal Medicine, Eastern Virginia Medical School, Norfolk, Virginia, United States of America; 3 Strelitz Diabetes Center, Eastern Virginia Medical School, Norfolk, Virginia, United States of America; NIDCR/NIH, UNITED STATES

## Abstract

Pathology driving β-cell loss in diabetes is poorly defined. Chronic subclinical inflammation is associated with β-cell dysfunction. Acute *in vitro* exposure of islets and β-cells to an inflammatory cytokine cocktail (IL-1β/TNF-α/IFN-γ) results in loss of cell function and viability. The contribution of each cytokine alone or in combination has been evaluated in homogeneous mouse β-cell lines and primary mouse islets. Cytokine cooperation is required for β-cell apoptosis with the most potent combinations including IL-1β. Single cytokine exposure did not induce β-cell apoptosis. Expression of endogenous interleukin-12 in β-cells correlated with inflammatory cytokine combinations that induced β-cell apoptosis. Uncoupling of the IL-12 axis by a block of IL-12 production, inhibition of IL-12 receptor/ligand interaction or disruption of IL-12 receptor signaling conferred protection to β-cells from apoptosis induced by inflammatory cytokine stimulation. Signaling through STAT4 is indicated since disruption of IL-12 concomitantly reduced inflammatory cytokine stimulation of endogenous IFN-γ expression. Primary mouse islets isolated from mice deficient in STAT4 show resistance to inflammatory-cytokine-induced cell death when compared to islets isolated from wild type mice. Collectively, the data identify IL-12 as an important mediator of inflammation induced β-cell apoptosis. Modulation of IL-12/STAT4 signaling may be a valuable therapeutic strategy to preserve islet/β-cell viability in established diabetes.

## Introduction

Worldwide diabetes incidence is predicted to exceed 592 million by 2035 [1]. Diabetes is a complex metabolic disease being influenced by numerous factors. A core feature is the failure of insulin producing β-cells for both type 1 (T1DM) and type 2 (T2DM) diabetes [2, 3]. Causes of β-cell failure are poorly understood, but chronic sub-clinical inflammation is a contributing factor. Inflammation is a feature of both T1DM and T2DM [4–12]. Acute exposure of islets to inflammatory cytokines *ex vivo* promotes islet stress and dysfunction, including loss of glucose-stimulated insulin secretion, increased apoptosis and elevated expression of various marker genes, including monocyte chemoattractant protein-1 (MCP-1) [13, 14]. Elevated MCP-1 in islets occurs during early insulitis in experimental diabetes mouse models and is used clinically to assess transplantable human islets [15]. Induction of islet dysfunction by inflammatory cytokines, especially the triple cytokine combination of IL-1β/TNF-α/IFN-γ, is extensively reported [16]. The cellular responses in islets and β-cells to inflammatory cytokine exposure are less well characterized.

Several cellular effects have been associated with exposure of β-cells to inflammatory cytokines [17, 18]. A candidate mediator of β-cell dysfunction is interleukin-12 (IL-12). Local production of IL-12 has been reported and may establish an islet:immune interface for targeted β-cell destruction [19]. IL-12, a heterodimeric ligand composed of subunits, p35 (IL-12 p35) and p40 (IL-12 p40), coordinates a Th1 immune response by inducing expression of IFN-γ. Principally considered an immune factor, IL-12 has also been identified in non-immune cells, including islets [19]. Being a key mediator in disease pathologies, several approaches to uncouple IL-12 action have been identified. STA-5326 (Apilimod®) is a small molecular weight compound that inhibits c-Rel translocation from the cytoplasm to the nucleus and disrupts transcription of both IL-12 p35 and IL-12 p40 [20–23]. Lisofylline (LSF) is a methylxanthine metabolite of Pentoxifylline that inhibits IL-12 signaling activity. LSF limits commitment to T-helper 1 cell development and IFN-γ production [24]. LSF stopped onset of Type 1 diabetes in NOD mice [25]. Antibodies that bind, sequester and neutralize IL-12 p40, eg Usterkinumab® and Briaknumab® have proven clinical efficacy in the autoimmune condition psoriasis [26–29]. Antibody-mediated neutralization of IL-12 p40 in islets conferred protection to β-cell dysfunction mediated by inflammatory cytokines [19]. Ligation of the IL-12 ligand to its heterodimeric receptor primarily activates (phosphorylates) signal transducer and activator of transcription 4 (STAT4). Genetic deletion studies show STAT4 is an important factor in elevating susceptibility to several autoimmune diseases. In terms of diabetes, NOD mice deficient in STAT4 do not develop spontaneous diabetes unlike wild-type NOD mice [30, 31].

Exposure of islet β-cells to pro-inflammatory cytokines results in β-cell dysfunction [14, 19]. The current report has identified a pivotal role for IL-12 and IL-12 mediated STAT4 signaling in the development of β-cell apoptosis. These data identify potential therapeutic targets for preservation of β-cell function and/or β-cell survival in established diabetes.

## Materials and Methods

### Ethics Statement and Mouse Islets

All protocols and procedures were performed in accordance with the “Principles of laboratory animal care” (NIH publication no. 85–23), AAALAC, and approved by Institutional Animal Care and Use Committee (IACUC protocol #11–013) at Eastern Virginia Medical School. Islets were isolated from C57BL/6J (Jackson Laboratory, Bar Harbor, ME) mice between the ages of 8 to 24 weeks by common bile duct cannulation and collagenase digestion [25]. STAT4ko mice (on C57BL/6J background; gift from Dr. Mark Kaplan, Indiana University) between the ages of 8 weeks to 28 weeks were used for islet isolation. Individual islets were hand picked and placed in 1 mL RPMI media (Life Technologies, Grand Island, NY) supplemented with 10% fetal bovine serum/antibiotics.

### Cell Line

βTC-3 (mouse) cells (as described in [32]) were cultured at 37°C with 5% CO_2_ in DMEM (Life Technologies) supplemented with 18% fetal bovine serum, 100units/mL penicillin, 100μg/mL streptomycin, 4mM L-glutamine, 25mM glucose, and 1mM sodium pyruvate.

### Treatment and apoptosis detection

Cell stimulation: Isolated islets or cells were treated for 24 hours with either a pro-inflammatory cocktail (PIC) of all three cytokines (IL-1β (5 ng/mL), TNF-α (10 ng/mL), and IFN-γ (100 ng/mL), R&D Systems, Minneapolis, MN), a combination of two of the cytokines, or an individual cytokine only. Selected inhibitors or antibody at the indicated concentrations (STA-5326 (1nM or 100nM) (Apilimod, Axon Medchem BV, Groningen, Netherlands); Lisofylline (LSF, 20μM); c47 (50μM); anti-IL-12 antibody (1μg/mL) (eBioscience, San Diego, CA)) were added 30 minutes prior to cytokine treatment and remained for the duration of treatment.

Fluorescence microscopy: Treated cells were washed in cold PBS and then incubated in cold PBS containing 0.1 μM YO-PRO-1 (Life Technologies) and 1 μg/mL Propidium Iodide (PI) at 4°C for 30 minutes. For cell lines, five random fields per well were analyzed and for islets, all islets were analyzed. The densitometric fluorescence value (green and red channels) were analyzed using ImageJ 1.42q (http://rsb.info.nih.gov/ij) to determine the fluorescence for each treatment. An apoptotic index was determined by relative expression of normalized signal with PIC being defined as unity. Images were captured with Axiophot (Zeiss, Jena, Germany) and Axiovision (Zeiss) image analysis.

Caspase-3 detection: Islets or cell line were treated for 4 hours without or with inhibitor. Cleavage of pro-caspase-3 was measured using a caspase-3 assay kit (BD Pharmigen, Franklin Lakes, NJ) according to the manufacturer’s instructions. Fluorescence was measured (SpectraMax, Molecular Devices, Sunnyvale, CA) using excitation wavelength 380 nm and emission wavelength 440 nm.

### Real-time PCR

Isolated islets from wild-type mice and STAT4ko mice or βTC-3 cells were treated with varying combinations of PICs for either 4 or 24 hours without or with inhibitor. After treatment, total RNA was isolated (RNeasy Mini Kit; Qiagen, Valencia, CA) and transcribed using murine leukemia virus reverse transcriptase (Life Technologies) and random hexamers (Life Technologies) using a 20μL reaction volume. RT-PCR was performed in a CFX96 Thermal Cycler (Bio-Rad, Hercules, CA). Total reaction volume was 25μL, which consisted of 3μL cDNA (five-fold dilution) and Jump Start *Taq* Polymerase (Sigma-Aldrich, St Louis, MO). The primers used with SYBR Green 1 (Molecular Probes, Carlsbad, CA) probes are shown in Table 1. Taqman primers were used for IL-12 p40, IL-12 p35, and IFN-γ (Life Technologies). All RT-PCR reactions were performed in triplicate. The housekeeping gene GAPDH was used to normalize the data. The 2^-ΔΔCT^ method was used to analyze the data.

**Table 1:** Primers used for RT-PCR.

Primer	Sequence
GAPDH forward	5’-TCA CCA CCA TGG AGA AGG C-3’
GAPDH reverse	5’-GCT AAG CAG TTG GTG GTG CA-3’
MCP-1 forward	5’-CTT CTG GGC CTG CTG TTC A-3’
MCP-1 reverse	5’-CCA GCC TAC TCA TTG GGA TCA-3’
IL-23 p19 forward	5’-CAG TCA GAG TTG CTG CTC CGT GG-3’
IL-23 p19 reverse	5’-CAG CCA ACT CCT CCA GCC AGA G-3’

### Data Analysis

Experiments were performed at a minimum in triplicate. For statistical analysis, student *t* test or one-way ANOVA with Tukey post hoc testing (Prism 4.0, Graph-Pad Software, La Jolla, CA) were used to determine statistical significance (95% CI and *p* < 0.05). Data set for analyses is provided (S1 Appendix).

## Results

### Induction of islet death by pro-inflammatory cytokines

**Fig 1:**
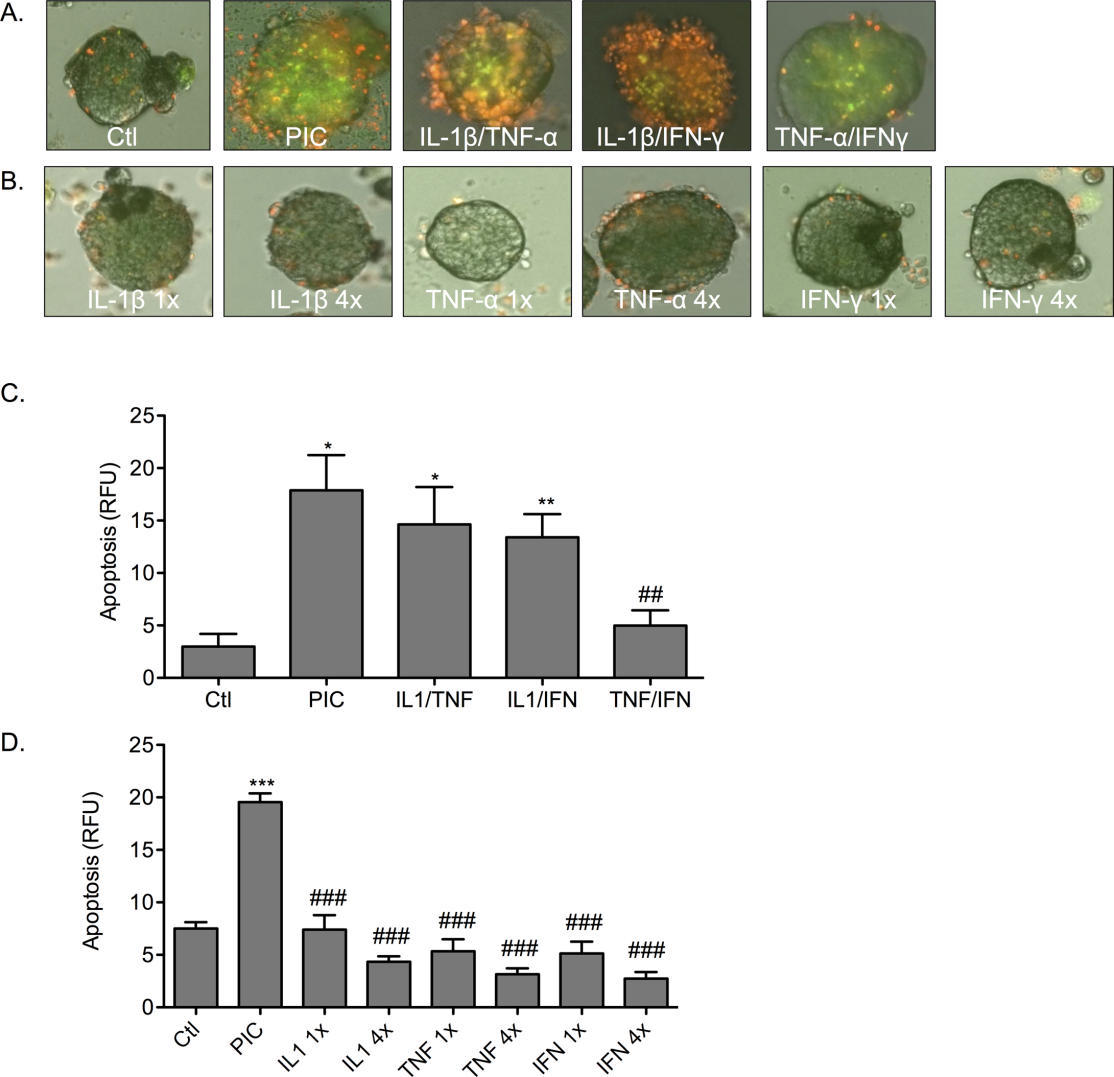
Single cytokine treatment is not sufficient to induce apoptosis in isolated mouse islets. Islets were examined microscopically following labeling with YO-PRO-1 (green) and propidium iodide (red). (A) Islets were treated with a triple cytokine cocktail of IL-1β/TNF-α/IFN-γ (PIC), or dual cytokine combinations of IL-1β/TNF-α, IL-1β/IFN-γ, or TNF-α/IFN-γ. (B) Islets were treated with a single cytokine of either IL-1β, TNF-α, or IFN-γ at a 1-fold (1x) or 4-fold (4x) dose. Graph shows quantified apoptosis (C) from dual cytokine treatment or (D) from single cytokine treatment for 1x and 4x doses for all islets per experiment * *p* < 0.05, ** *p* < 0.01, *** *p* < 0.001 relative to Ctl, ## *p* < 0.01, ### *p* < 0.001 relative to PIC and n = 3.

Acute overnight treatment of isolated mouse islets with a cocktail of pro-inflammatory cytokines (PIC; IL-1β/TNF-α/IFN-γ) induced cell death (Fig 1). A significant increase in cell death in PIC-treated islets was detected using the fluorescent viability dyes YO-PRO-1 (green) and propidium iodide (red) when compared to non-cytokine treated control (Ctl) islets. Triple cytokine treatment (PIC) induced a significant increase in fluorescence when compared to untreated islets (*p* < 0.05). Representative images are shown in Fig 1A. The data is quantified in Fig 1C. Relative fluorescence units (RFU) for triple cytokine treated islets was 17.9 ± 3.3 RFU as compared to 2.9 ± 1.2 RFU for control islets.

To determine if individual cytokine exposure was sufficient for the induction of islet death, islets were treated with single cytokines. Representative images are shown in Fig 1B. The data is quantified in Fig 1D. Islets were incubated with each cytokine at the equivalent dose used in the PIC cocktail (one-fold; 1X) and at four-fold this dose (4X) (Fig 1B and 1D). Incubation either with IL-1β, TNF-α, or IFN-γ alone (1X) did not induce elevated cell death relative to control islets. Increasing the dose of each cytokine 4-fold did not significantly induce cell death. Islets that were treated with single cytokines at either a 1X or 4X dose showed significantly less cell death than islets treated with PICs (*p* < 0.001) and no significant difference to control (Fig 1D).

Paired combinations of the cytokines IL-1β, TNF-α, and IFN-γ were used to determine if a dual cytokine treatment was sufficient to induce islet death equivalent to PIC treatment. Significant increases in cell death (fluorescence) were observed and quantitated when the cytokine combinations of IL-1β/TNF-α and IL-1β/IFN-γ were used to treat islets (*p* < 0.05, *p* < 0.01; relative to control; Fig 1A and 1C). Treatment with the combination TNF-α/IFN-γ did not significantly increase cell death when compared to control islets (ctl 2.9 ± 1.2 RFU; n = 3). Results from quantitative analyses of fluorescence (Fig 1C) showed that cell death for triple cytokine treated islets (17.9 ± 3.3 RFU) was greater than IL-1β/TNF-α (14.6 ± 3.6 RFU), IL-1β/IFN-γ (13.4 ± 2.2 RFU), and TNF-α/IFN-γ (4.9 ± 1.4 RFU) treated mouse islets respectively. The dual cytokine treatment of TNF-α/IFN-γ induced cell death significantly less than triple cytokine (PIC)-treatment (*p* < 0.01).

Triple cytokine (PIC)-treatment is a more efficacious inducer of cell death in islets compared to treatment either with a single cytokine or dual combination of these pro-inflammatory cytokines.

### Effect of pro-inflammatory cytokine treatment on MCP-1 gene expression

**Fig 2:**
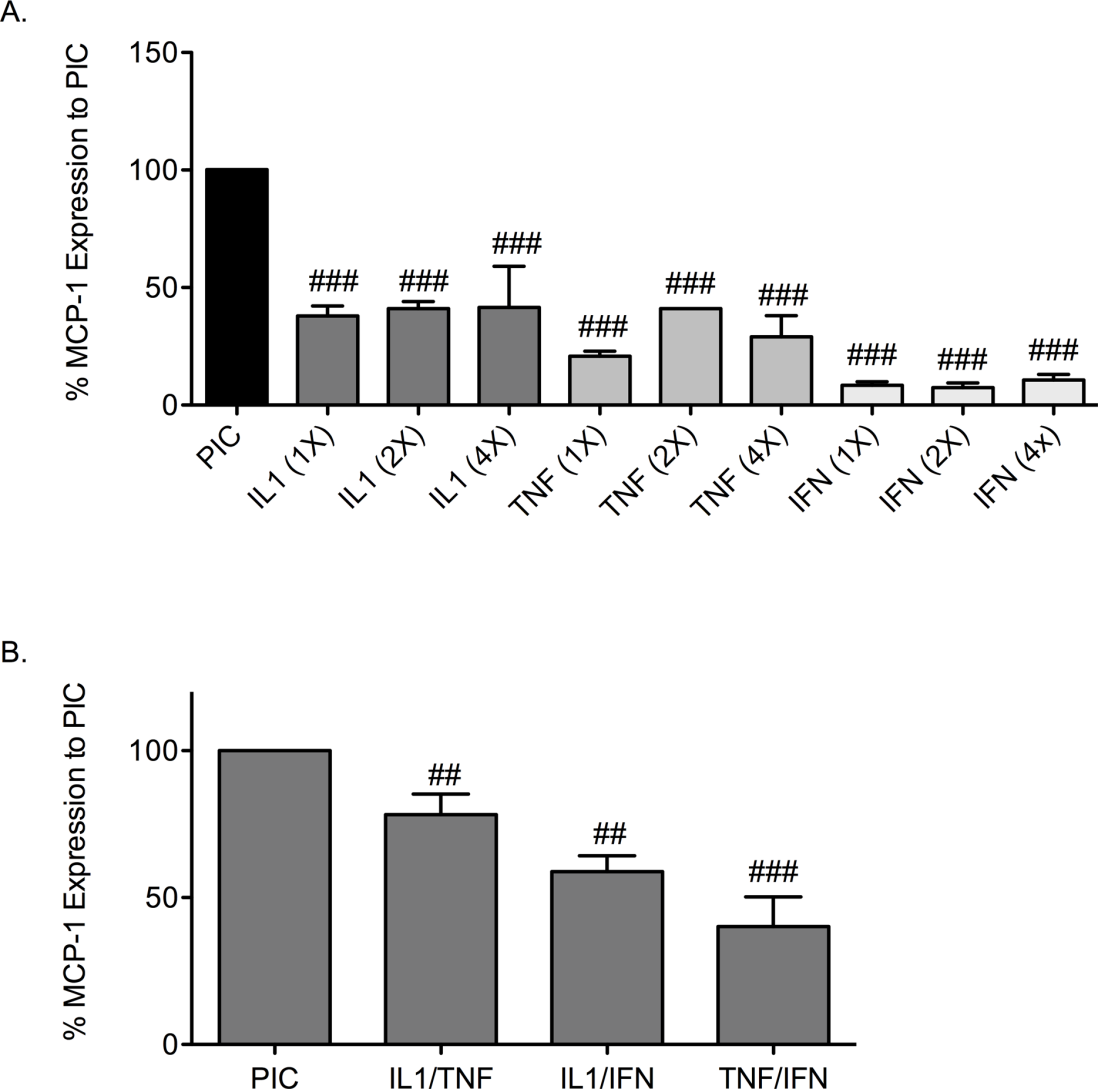
Cytokine induced MCP-1 in mouse islets. Expression of MCP-1 gene following stimulation of mouse islets with single cytokines (A) IL-1β, TNF-α, or IFN-γ at 1x, 2x, or 4x or dual cytokines with (B) IL-1β/TNF-α, IL-1β/IFN-γ, or TNF-α/IFN-γ relative to stimulation with PICs. ## *p* < 0.01, ### *p* < 0.001 relative to PIC and n = 3.

MCP-1 is a chemokine that is released from islets under conditions of stress and is a recognized marker of β-cell stress. To determine if cytokine-induced changes in MCP-1 corresponded with induction of cell death in mouse islets, expression of MCP-1 gene was measured. The greatest increase in MCP-1 expression occurred in mouse islets treated with triple cytokines (PIC; Fig 2A). Relative to MCP-1 following PIC-treatment (defined as 100%) the expression of MCP-1 by single cytokines at 4-fold concentration was 41.5% ± 17.5 IL-1β, 29% ± 9 TNF-α, and 10.7% ± 2.3 IFN-γ. Expression of MCP-1 was significantly less than PICs in islets treated with single cytokine at 1-fold, 2-fold, and 4-fold doses (*p* < 0.001).

MCP-1 expression was determined in mouse islets treated with paired combinations of cytokines (Fig 2B). Induction of MCP-1 gene expression by IL-1β/TNF-α, IL-1β/IFN-γ, and TNFα/IFNγ was significantly less than PIC-treated islets (*p* < 0.01, *p* < 0.01, *p* < 0.001). IL-1β/TNF-α, IL-1β/IFN-γ, and TNF-α/IFN-γ stimulated relative MCP-1 expression 78% ± 7.0, 59% ± 5.4, and 40% ± 10 of the induction seen with triple cytokines (defined as 100% expression). These data indicate that the cytokine combinations IL-1β/TNF-α, IL-1β/IFN-γ, and TNF-α/IFN-γ are able to induce MCP-1 gene expression although are less effective than triple cytokine treatment (Fig 2). These data correlate with the cell death studies (Fig 1).

### Pro-inflammatory cytokine treatment induces IL-12 gene expression

**Fig 3:**
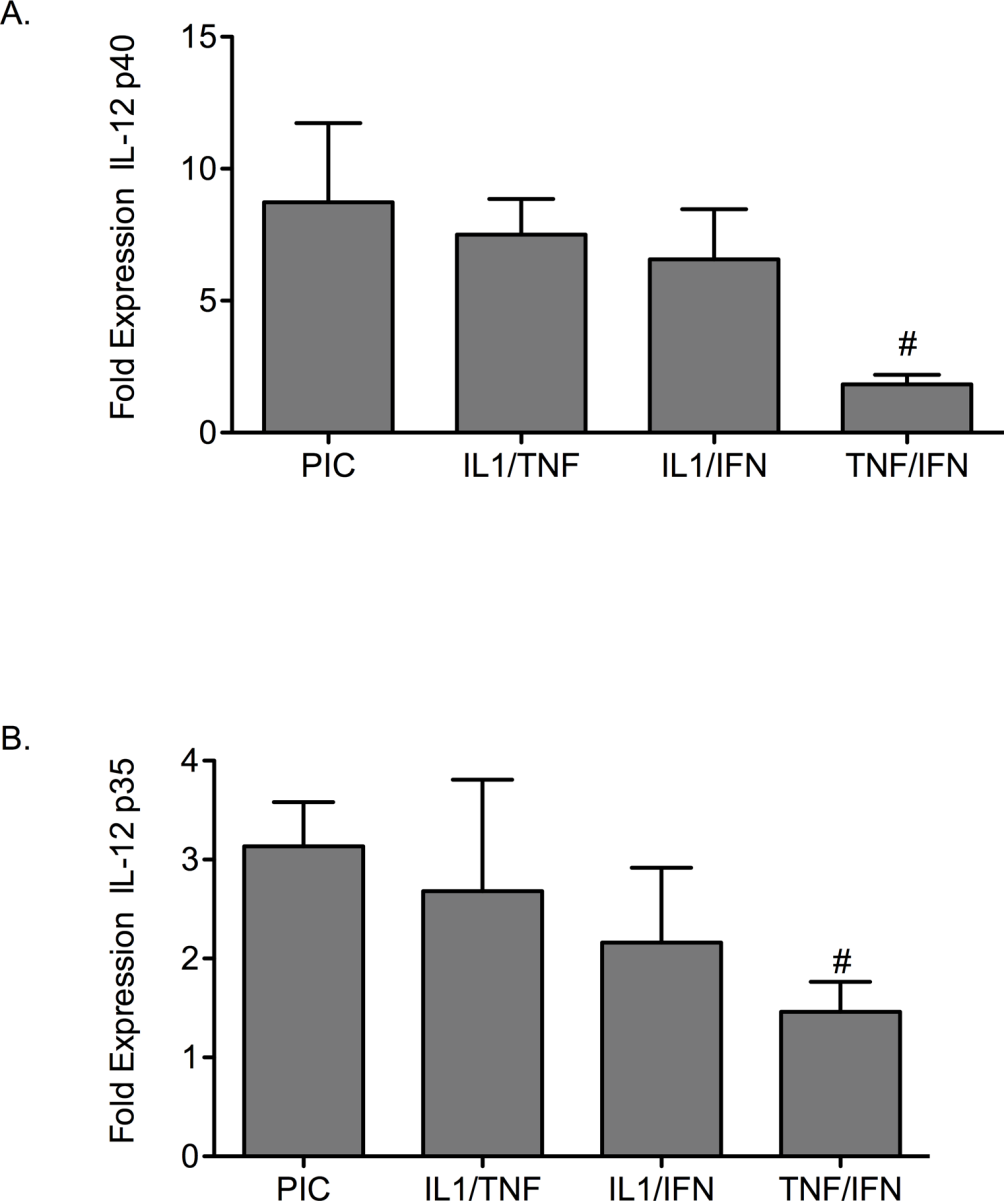
Cytokine treatment affects IL-12 gene expression in mouse islets. (A) IL-12 p40 and (B) IL-12 p35 gene expression was measured in islets treated with PICs or the dual cytokine combinations of IL-1β/TNF-α, IL-1β/IFN-γ, or TNF-α/IFN-γ. # *p* < 0.05 relative to PIC and n = 3.

A candidate pathway in the development of β-cell dysfunction in diabetes is expression and production of IL-12 and IL-12 mediated signaling. To determine the influence of pro-inflammatory cytokine treatment on the IL-12 pathway, gene expression for the IL-12 ligand (IL-12 p40 and IL-12 p35) in response to cytokine combinations was tested in mouse islets (Fig 3). Dual cytokine combinations of IL-1β/TNF-α and IL-1β/IFN-γ induced IL-12 p40 and IL-12 p35 gene expression to levels equivalent to those induced by PIC-stimulation (Fig 3A and 3B). Induction of IL-12 genes with TNF-α/IFN-γ treatment was significantly less than the other dual cytokine treatments or triple cytokine treatment (*p* < 0.05).

**Fig 4:**
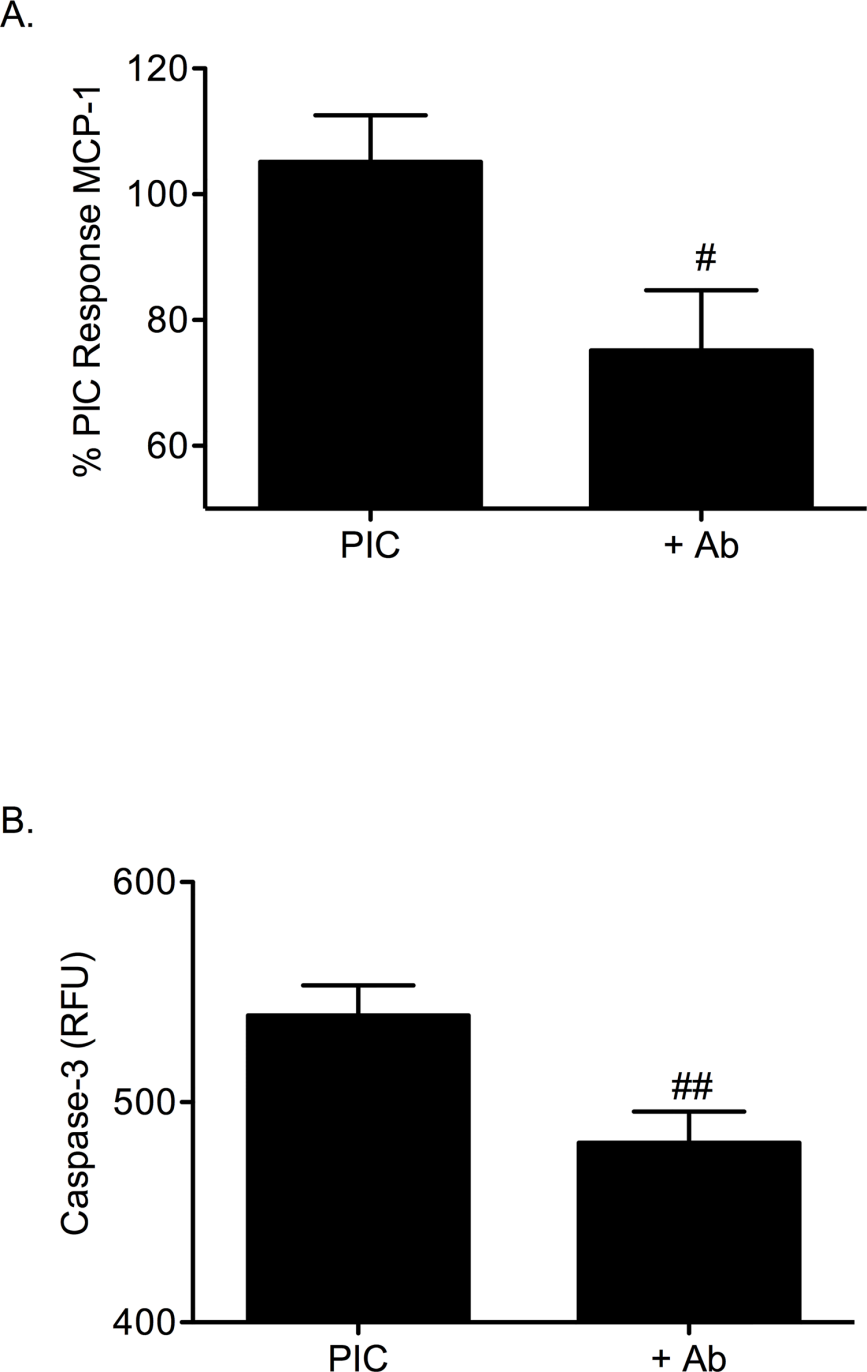
A neutralizing antibody to IL-12 p40 protects β-cells from PIC-induced apoptosis. (A) MCP-1 gene expression in PIC-treated βTC-3 cells without or with IL-12 p40 neutralizing antibody. (B) Caspase-3 activity in PIC-treated βTC-3 cells without or with IL-12 p40 neutralizing antibody. Graph (B) shows pro-caspase-3 cleavage (RFU). # *p* < 0.05, ## *p* < 0.01 relative to PIC and n = 3.

To support the role of the IL-12 pathway in β-cell dysfunction, a neutralizing antibody to the IL-12 p40 ligand was used to disrupt IL-12. MCP-1 gene expression and apoptosis were examined in the homogenous mouse β-cell line βTC-3s (Fig 4). Apoptosis was measured by caspase-3 cleavage. When βTC-3 cells were treated overnight with PICs and a neutralizing IL-12 p40 antibody (1μg/mL), expression of MCP-1 gene was significantly decreased by 27% ± 5.0 (*p* < 0.05, Fig 4A). Addition of the IL-12 neutralizing antibody significantly decreased PIC-induced caspase-3 activity in βTC-3s (539 ± 14 RFU to 482 ± 14 after subtraction of background 226.3 ± 5.7 RFU) (*p <* 0.01, Fig 4B).

### Inhibition of the IL-12 pathway confers protection in PIC-induced β-cell apoptosis

**Fig 5:**
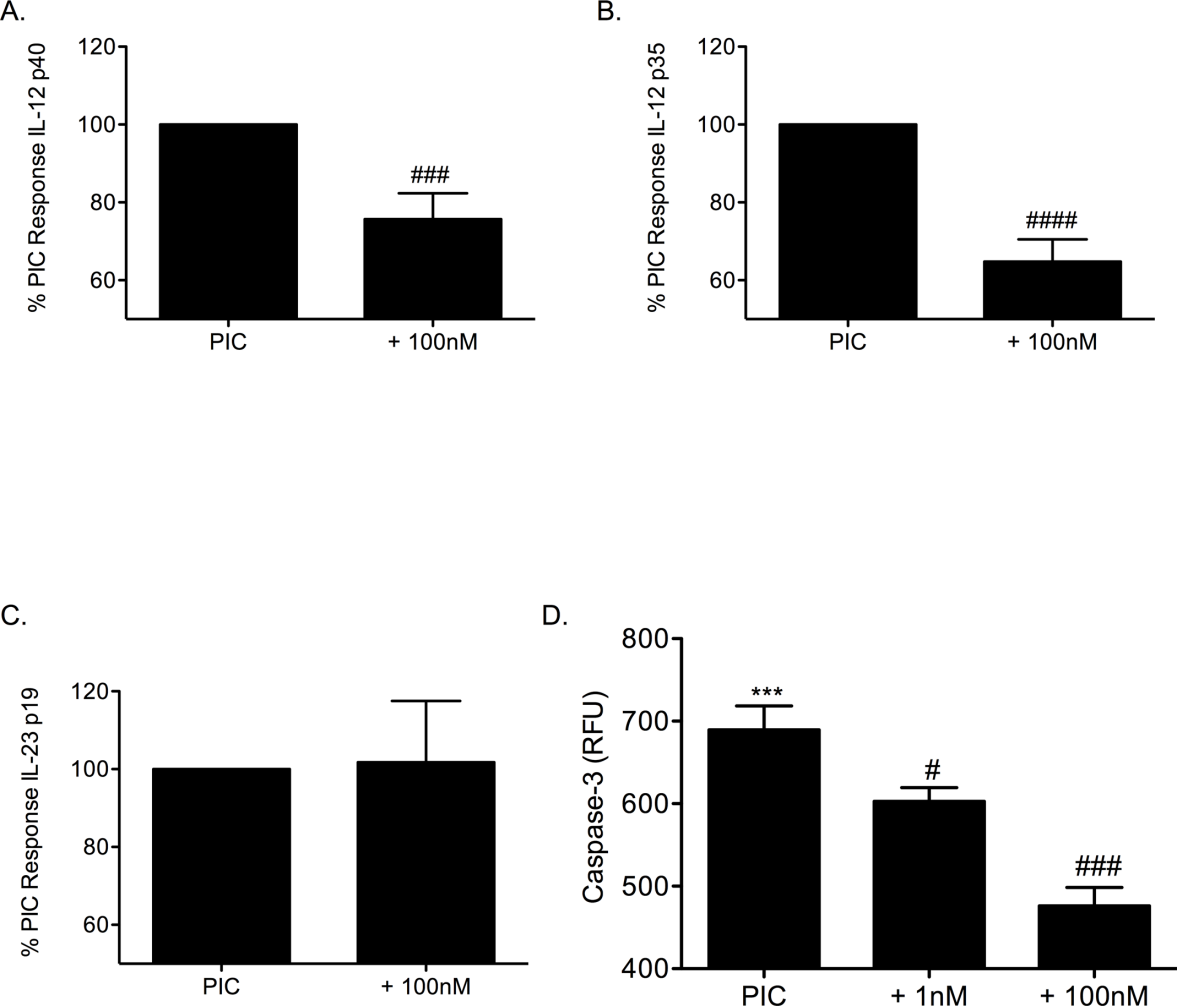
STA-5326 inhibits PIC-induced IL-12 gene expression in β-cells and improves β-cell survival. (A) IL-12 p40, (B) IL-12 p35, and (C) IL-23 p19 gene expression in βTC-3 cells treated with PICs in the absence or presence of 100nM STA-5326. (D) Pro-caspase-3 cleavage in PIC-treated βTC-3 cells without or with 1nM or 100nM STA-5326. *** *p* < 0.001 relative to Ctl, # *p* < 0.05, ### *p* < 0.001, #### *p* < 0.0001 relative to PIC and n = 3.

A transcriptional inhibitor of IL-12 gene activity was used to explore the effect of IL-12 on β-cell survival and gene expression. STA-5326 inhibits gene expression of IL-12 p40 and IL-12 p35. Addition of 100nM STA-5326 to PIC-treated βTC-3 cells significantly decreased PIC-induced IL-12 p40 (Fig 5A) and IL-12 p35 (Fig 5B) gene expression (25% ± 6.6 and 35% ± 5.7 respectively of PIC-induced levels; *p* < 0.001, *p* < 0.0001). βTC-3 cells treated with PICs and STA-5326 did not show a significant decrease in IL-23 p19 gene expression (Fig 5C). STA-5326 significantly decreased caspase-3 activity in βTC-3 cells treated with PICs (Fig 5D: PIC, 689.5 ± 29 RFU; PIC + 1nM STA-5326, 602.7 ± 17 RFU; PIC + 100nM STA-5326, 476.2 ± 22 RFU; *p* < 0.05, *p* < 0.001).

**Fig 6:**
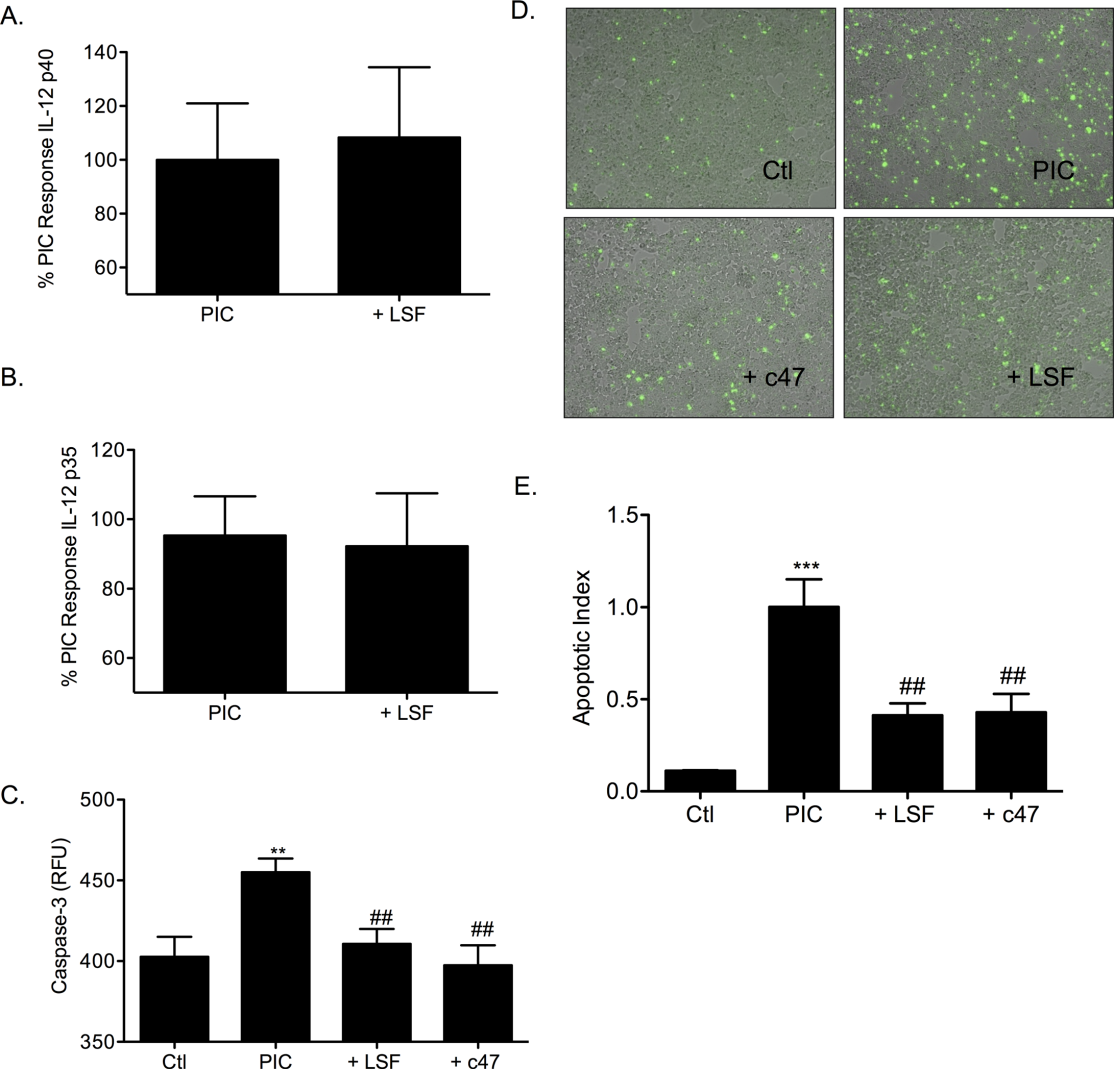
Lisofylline protects β-cells from PIC-induced apoptosis. (A) IL-12 p40 and (B) IL-12 p35 gene expression in PIC-treated βTC-3 cells without or with 20μM Lisofylline (LSF). (C) PIC-induced caspase-3 activation in the absence or presence of 20μM LSF or 50μM c47 in βTC-3 cells. Graph shows pro-caspase-3 cleavage relative fluorescent units (RFU). (D) Apoptosis was measured in PIC-treated βTC-3 cells without or with 20μM LSF or 50μM c47. Cells were examined microscopically following labeling with the viability dye, YO-PRO-1 (green). Representative images are shown for untreated (Ctl), PIC-treated, PIC with 50μM c47, or PIC with 20μM LSF respectively. (E) Graph shows quantified apoptotic index. ** *p* < 0.01, *** *p* < 0.001 relative to ctl, ## *p* < 0.01 relative to PIC and n ≥ 3.

Additional candidate inhibitors of the IL-12 pathway were also tested in βTC-3 cells and mouse islets to determine the importance of this pathway in β-cell survival. Lisofylline (LSF) inhibits the activity of IL-12. LSF and an efficacious structural analog c47 were used to determine if inhibition of the IL-12 pathway confers β-cell survival. Addition of 20μM LSF prior to PIC treatment in βTC-3 cells did not change the levels of PIC-induced IL-12 p40 and p35 gene expression (Fig 6A and 6B). Caspase-3 activity was significantly increased with PIC-treatment of βTC-3 cells (455.1 ± 8.5 RFU; *p <* 0.01) when compared to untreated control cells (402.7 ± 12 RFU) (Fig 6C). Addition of either LSF or c47 prior to PIC-treatment significantly decreased apoptosis observed in cells (PIC + 20μM LSF 410.7 ± 9.2 RFU, PIC + 50μM c47 397.4 ± 12.4 RFU; *p <* 0.01, *p <* 0.01). Detection of cell death by fluorescence (YO-PRO-1) showed LSF and c47 significantly protecting β-cells from PIC-induced cell death (*p <* 0.01). Representative images and quantitation are shown in Fig 6D and 6E respectively.

**Fig 7:**
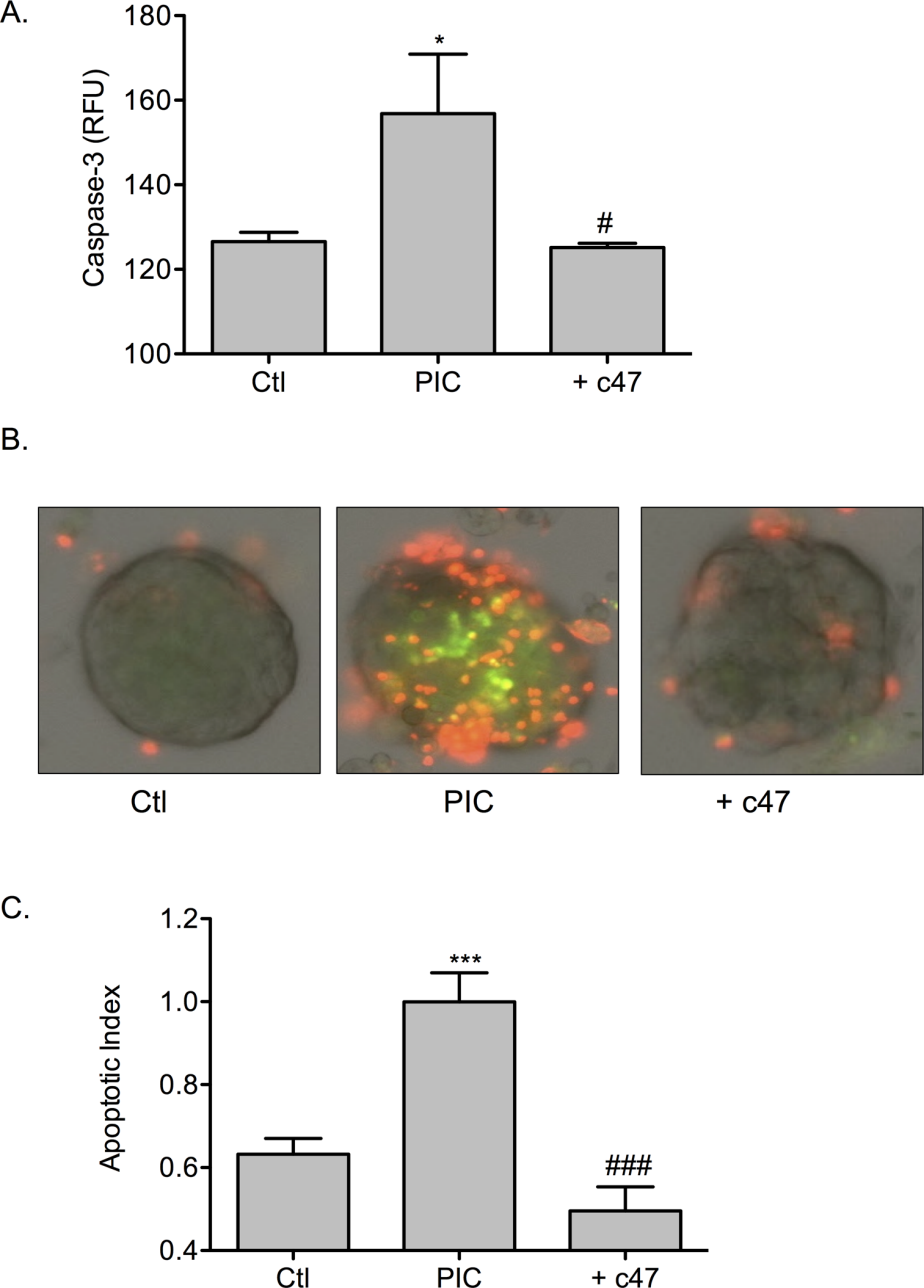
c47 confers protection to mouse islets from PIC-induced apoptosis. (A) PIC-induced caspase-3 activation in the absence or presence of 50μM c47 in mouse islets. Graph shows pro-caspase-3 cleavage. (B) Apoptosis was measured in PIC-treated islets without or with 50μM c47. Cells were examined microscopically following labeling with YO-PRO-1 (green) and PI (red). Representative images are shown for untreated (Ctl), PIC-treated, or PIC with 50μM c47 respectively. (C) Graph shows quantified apoptotic index. * *p* < 0.05, *** *p* < 0.001 relative to ctl, # *p* < 0.05, ### *p* < 0.001 relative to PIC and n > 3.

Inhibitor c47 was used to disrupt the IL-12 pathway in mouse islets. Mouse islets treated with PICs showed a significant increase in β-cell apoptosis measured by caspase-3 cleavage relative to untreated (Ctl) islets (Fig 7A; PIC, 156.9 ± 14.1 RFU vs Ctl, 132.1 ± 5.8 RFU; *p* < 0.05). Following addition of c47, PIC-induced caspase-3 cleavage was significantly decreased (PIC + c47, 132.3 ± 3.1 RFU; *p* < 0.05). Cell death was additionally measured by fluorescence. Preserved β-cell survival was observed when mouse islets were treated with PICs and c47. Representative images of fluorescent tagged cell death and quantitation of apoptotic index are shown in Fig 7B and 7C respectively. Measurement of fluorescence showed PIC-treatment significantly increased apoptosis of islets (*p* < 0.001). Consistent with the caspase-3 study, addition of c47 to PIC stimulation significantly decreased PIC-induced apoptosis (Fig 7C; *p* < 0.001).

Inhibition of the IL-12 pathway through transcriptional down-regulation of IL-12 p40 and IL-12 p35 by STA-5326 or inhibition of IL-12 activity through LSF or c47 leads to a decrease in PIC-induced β-cell/islet death.

### STAT4 signaling is a key component of the IL-12 pathway in PIC-induced islet death

**Fig 8:**
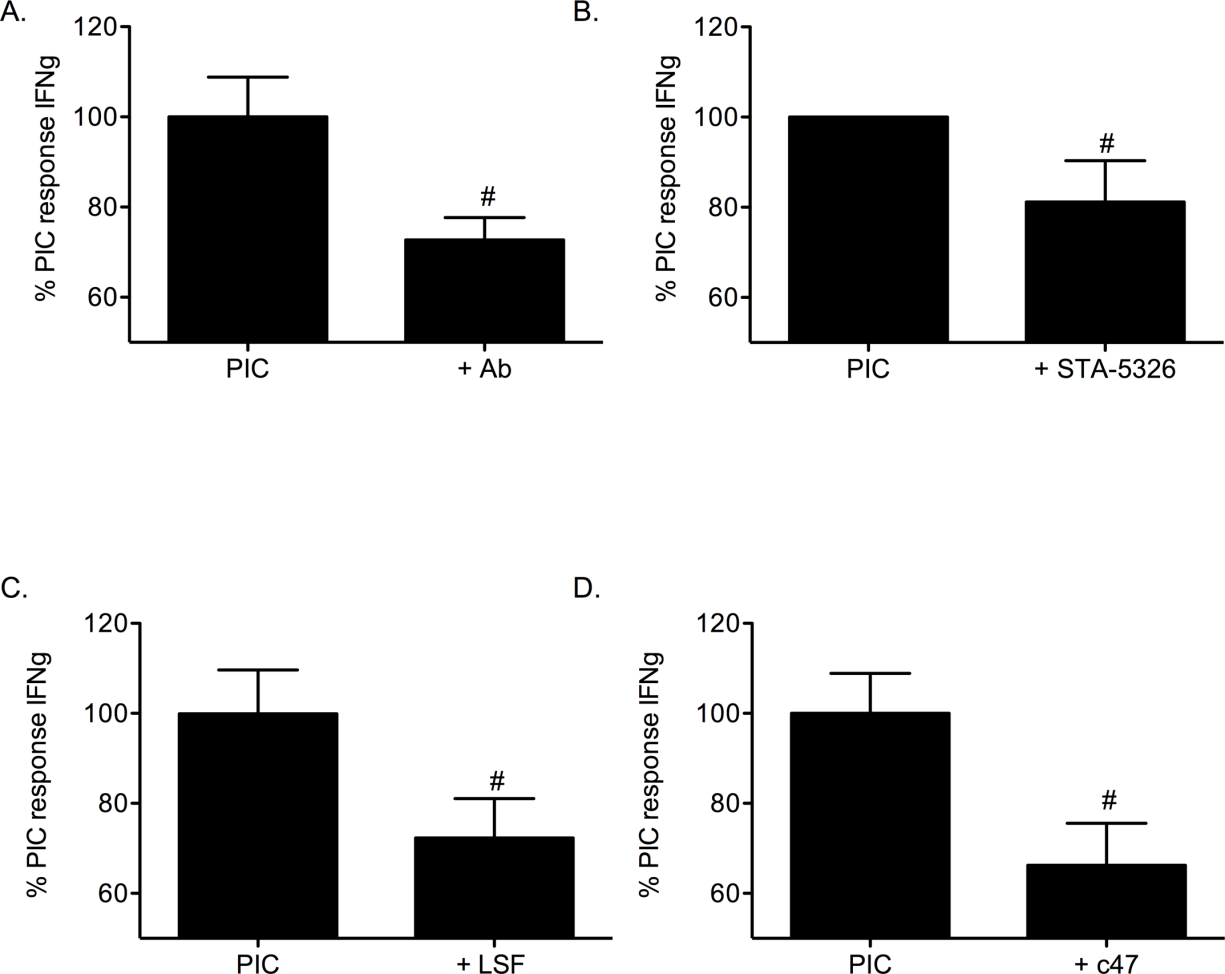
Inhibition of the IL-12 pathway reduces PIC-induced IFN-γ gene expression in β-cells. The β-cell line βTC-3 was treated with PICs in the absence or presence of (A) 1μg/mL IL-12 p40 neutralizing antibody, (B) 100nM STA-5326, (C) 20μM LSF, or (D) 50μM c47 prior to determination of IFN-γ gene expression. # *p* < 0.05 relative to PIC and n = 3.

A characteristic consequence of IL-12 ligand/receptor ligation is expression of IFN-γ. To examine whether IL-12 induces IFN-γ gene expression in β-cells agents that uncouple IL-12 (IL-12 p40 neutralizing antibody, STA-5326, LSF, or c47) were tested in the presence of PICs (Fig 8A–8D). PIC-stimulation of the mouse β-cell line, βTC-3 induced IFN-γ expression. Expression of the IFN-γ gene by PIC-treatment (defined as 100%) was significantly reduced with inclusion of either the IL-12 p40 neutralizing antibody (27% ± 5.0%), STA-5326 (19% ± 9.1%), LSF (28% ± 8.8%), or c47 (34% ± 9.4%) (*p <* 0.05).

**Fig 9:**
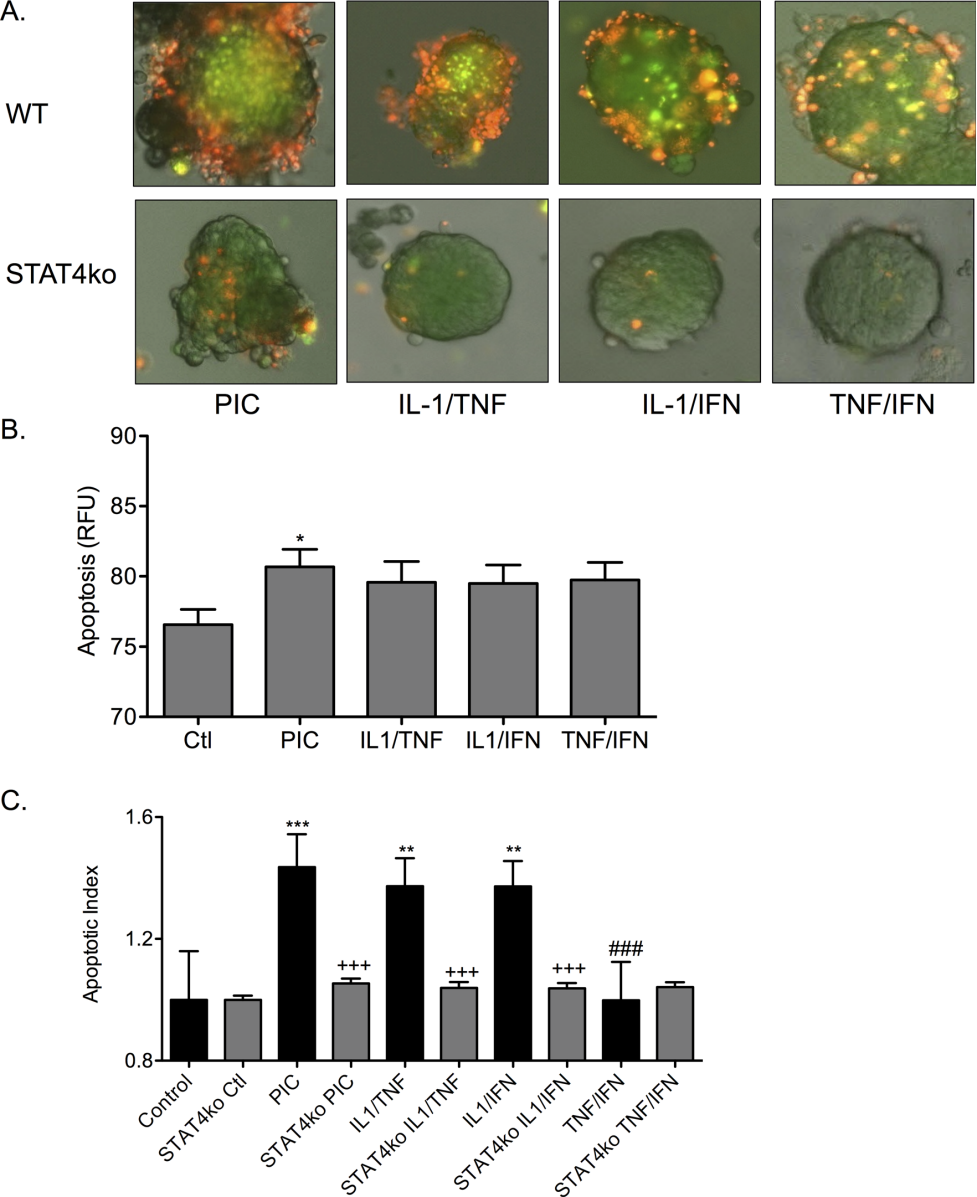
Islets from STAT4 deficient mice are resistant to PIC-induced apoptosis. (A) Fluorescence microscopy was used to image apoptotic islets labeled with YO-PRO-1 (green) and PI (red). Islets from WT mice (top row) or islets from STAT4ko mice (bottom row) were treated with PICs, IL-1β/TNF-α, IL-1β/IFN-γ, or TNF-α/IFN-γ. (B) Graph shows quantified apoptosis from cytokine treated STAT4ko islets. (C) Graph shows quantified apoptotic index from cytokine treated WT islets vs STAT4ko islets. * *p* < 0.05, ** *p* < 0.01, *** *p* < 0.001 relative to ctl, +++ *p* < 0.001, ### *p* < 0.001 relative to WT PIC treatment. Three groups of each condition were treated and analyzed.

STAT4 is a second messenger activated by IL-12 receptor ligation that transcriptionally activates the IFN-γ gene. To determine if STAT4 signaling is important for cytokine-induced islet apoptosis, the effect of cytokine combinations was compared between wild-type islets and islets from mice genetically deficient in STAT4 (STAT4ko islets). Cell death in mouse islets was determined by fluorescence (representative images in Fig 9A). Islet death occurred in WT islets treated overnight either with PICs (1.44 ± 0.11 RFU), IL-1β/TNF-α (1.37 ± 0.09 RFU), and IL-1β/IFN-γ (1.37 ± 0.08 RFU) relative to control (1.00 ± 0.16 RFU) (Fig 9C). Fluorescent intensity was measured for each treatment in the STAT4ko islets and plotted (Fig 9B). Following treatment with PICs, cell death in STAT4ko islets was significantly less than in PIC-treated WT islets (*p* < 0.001). Cell death that was observed in the STAT4ko islets treated with the dual cytokine combinations was not significantly different to control. Comparing islets from WT mice to STAT4ko mice, islets that lacked STAT4 were protected from apoptosis after treatment with PICs, IL-1β/TNF-α, and IL-1β/IFN-γ (*p* < 0.001) (Fig 9C). Treatment with TNF-α/IFN-γ did not induce significant apoptosis in either WT islets or STAT4ko islets. Disruption of STAT4 signaling protected islets from apoptosis induced by PICs, and the dual-cytokine combinations of IL-1β/TNF-α and IL-1β/IFN-γ.

Collectively, triple cytokine treatment and the dual cytokine treatments of IL-1β/TNF-α and IL-1β/IFN-γ significantly induce β-cell death. These pathways involve IL-12. Triple cytokines (IL-1β/TNF-α/ IFN-γ), IL-1β/TNF-α and IL-1β/IFN-γ but not TNF-α/IFN-γ induce IL-12 p40 and IL-12 p35 gene expression. By uncoupling the IL-12 pathway, PIC-treated β-cells are protected from PIC-induced cell death. Islets from STAT4ko mice are resistant to cytokine-induced cell death relative to WT mouse islets.

## Discussion

This study has explored the relationship of cytokine soluble mediators elevated in inflammation to β-cell dysfunction. Specifically, identification of pathways key to inflammation-mediated β-cell apoptosis have been made. Induced β-cell dysfunction following acute exposure to a triple cytokine cocktail (IL-1β/TNF-α/IFN-γ) has been extensively reported (reviewed in [4];[33]). Inflammation is an increasingly recognized feature of diabetes pathogenesis [34–36]. Clinical focus has recently concentrated on the contribution IL-1β to β-cell dysfunction. A number of approaches have aimed to neutralize IL-1β in a bid to assess the therapeutic effectiveness in human diabetes [37–39].

The relative efficacy for inflammatory cytokines, in combination, to induce β-cell apoptosis have been assessed using the model systems of this study. The cytokine cocktail of IL-1β/TNF-α/IFN-γ effectively induced β-cell dysfunction/death. Each single cytokine alone at equivalent concentration, or up to four-fold that used in the triple cocktail, did not induce β-cell apoptosis. These data suggest that second messengers specific for each cytokine combine to induce β-cell death rather than a mere stimulation of a critical density in common signaling pathways. Dual cytokine combinations showed an apparent hierarchy for induction of β-cell apoptosis such that pairs including IL-1β (IL-1β/TNF-α or IL-1β/IFN-γ) were more potent than the combination of TNF-α/IFN-γ. Paired cytokines were less effective than triple cytokines. These results support an important role for IL-1β for induction of β-cell dysfunction and death while additionally highlighting the necessitated contribution of other inflammatory factors. As a therapeutic in diabetes, reduction of IL-1β, either by soluble receptor antagonism or antibody sequestration and neutralization was not effective in human clinical trials [40].

The cytokine interleukin-12 (IL-12) has been shown to be upregulated in β-cells following exposure to triple cytokines [19]. Exogenous IL-12 stimulated β-cell dysfunction and death [19]. IL-12 is a heterodimeric ligand formed by two protein chains IL-12 p40 and IL-12 p35. A heterodimer of IL-12 p40 and a distinct protein chain IL-23 p19, forms functional interleukin-23 (IL-23). The effect of dual cytokine stimulation was assessed on expression of IL-12 chains in β-cells. Dual cytokine pairings that included IL-1β induced expression of IL-12 to levels comparable to triple cytokines. Induction of IL-12 with TNF-α/IFN-γ was significantly lower. Overall the pattern of IL-12 expression by inflammatory cytokine combinations conformed with induction of cell death. These data show IL-12 expression is concomitant with cell death and suggested the importance of IL-12 as a mediator of cell death induced by the three cytokines. The studies in this report further explore the role of IL-12 and IL-12 mediated signaling in beta cell apoptosis. Activation of the IL-12 axis was interrupted in three separate approaches; inhibition of IL-12 ligand expression, inhibition of IL-12 receptor ligation and inhibition of IL-12 signaling. Disruption of IL-12 was effective at protecting β-cells from apoptosis associated with inflammatory cytokine stimulation.

Expression of IL-12 has been linked with autoimmune diabetes. In NOD mice, IL-12 plays a significant role in the transition from non-destructive to destructive insulitis [41]. In human type 1 diabetes (TIDM), the genetic susceptibility locus, IDDM18, is located near a regulatory allele of the IL-12 p40 gene (IL-12B) [42], and single nucleotide polymorphisms in this region associate with an earlier age of T1DM onset and accelerated deterioration in glycemic control [43, 44]. Systemic daily administration of IL-12 to NOD mice increased T1DM incidence whereas addition of an IL-12 antagonist decreased T1DM incidence [41, 45, 46]. During the development of diabetes in NOD mice and BB rats the expression of endogenous IL-12 p40 and IFN-γ increase prior to diabetes onset [47, 48]. To address if local expression of IL-12 in β-cells could initiate diabetes, Holtz et al, generated transgenic mice expressing IL-12, or monomer IL-12 chains in the β-cell [49]. RIP-IL-12, but not monomer–p35 or–p40, mice developed pancreatic islet inflammation. This was associated with an elevation in IFN-γ. Transgenic deletion of IL-12 or IFN-γ did not fully protect against diabetes development [50, 51]. Thus, additional pathways may contribute to β-cell dysfunction or mechanisms that are independent of IL-12 and IFN-γ may develop as a homeostatic response in transgenic mice lacking IL-12 or IFN-γ [51–53]. Studies with the IL-12 natural antagonist (p40)_2_ identified a key role for IL-12 in the development of T1DM [45, 46, 54].

The model systems studied indicate that intracellular signaling resulting from IL-12/IL-12-receptor ligation contribute to PIC-induced β-cell death. This conclusion was based on the fact that STA-5326, an inhibitor of c-Rel translocation that blocks transcriptional activation of IL-12 genes, reduced expression of IL-12 ligand chains but not the IL-23-specific chain p19 [20, 21, 23]; second, Lisofylline, an inhibitor of IL-12 signaling, did not inhibit production of IL-12 protein chains.

A primary response of IL-12/IL-12 receptor ligation is induction of IFN-γ expression. The three cytokine cocktail (PIC) induced endogenous IFN-γ gene expression in β-cells. Cytokine induction of IFN-γ in β-cells and islets has previously been reported [19]. The three approaches used to disrupt IL-12 in this study each blocked production of endogenous IFN-γ gene expression associated with PIC stimulation.

A principle signaling pathway linking IL-12 receptor ligation and expression of IFN-γ is activation of STAT4 [55]. Studies in STAT4-deficient mice show that STAT4-mediated IL-12-signaling regulates IFN-γ production and the generation of Th1 responses [55]. Pro-inflammatory cytokines are stimulators of IL-12 expression [56, 57]. Elevation in serum pro-inflammatory cytokines is a feature of both TIDM and T2DM [4–12]. The importance of STAT4 signaling in PIC-induced islet death was demonstrated. Islets from STAT4 deficient mice were protected to the effects of PICs when compared to islets from wild type mice. Inhibition of STAT4 may be an approach to more selectively target IL-12 unlike strategies to neutralize/sequester IL-12p40 that impact both IL-12 and IL-23 [31]. Human polymorphisms link STAT4 to autoimmune disorders including T1DM [58–61]. In experimental diabetes, a genetic disruption of STAT4 activation prevented the spontaneous development of diabetes in NOD mice [30, 31].

In summary, this study supports the susceptibility of islet β-cells to inflammation and highlights synergy with inflammatory cytokine combinations, especially those paired with IL-1β. A key event in inflammation induced β-cell death is IL-12 receptor ligation. Blockade of the IL-12 axis preserve β-cells from inflammatory-cytokine induced cell death. Activation of STAT4 is implicated. Agents that disrupt IL-12 signaling or STAT4 signaling may be effective therapeutic tools to prevent or treat diabetes.

## Supporting Information

S1 Appendix The dataset for the reported results is provided as supporting information per PLOS ONE policy. (PDF)
