# Supplementary material for: Interleukin-12 (IL-12)/STAT4 Axis Is an Important Element for β-Cell Dysfunction Induced by Inflammatory Cytokines
Source: PLoS One. 2015 Nov 10;10(11):e0142735. doi: 10.1371/journal.pone.0142735 (PMC4640700; doi:10.1371/journal.pone.0142735)
Supplement: S1 Appendix — (PDF) [file pone.0142735.s001.pdf]

Fig 1C

Ctl	PIC	IL1/TNF	IL1/IFN	TNF/IFN
1	34.642	11.018	10.58	11.793
1	19.723	16.797	32.632	11.622
1	31.527	6.017	14.68	1
7.16	33.42	1	22.947	1
1.861	39.934	39.934	1	14.412
1	9.849	1	5.541	15.919
1	3.083	9.849	16.26	1
9.667	43.857	3.083	8.644	0.112
	7.834	43.857	1.797	1
	9.609	7.834	24.768	23.284
	12.45	14.297	16.09	11.02
	6.386	33.182	20.097	1.881
	8.168	7.261	18.784	1.449
	7.591	7.119	23.547	2.132
	4.943	30.81	11.611	1
	25.048	1	10.413	1
	6.16		1	1
			1	1
				1
				1
				1

Fig 2A

PIC	IL1 (1X)	IL1 (2X)	IL1 (4X)	TNF (1X)	TNF (2X)	TNF (4X)	IFN (1X)	IFN (2X)	IFN (4x)
100.01	31	38	59	26	41	N/D	8.6	5.5	N/D
100	32	44	N/D	19	N/D	20	11	9.4	8.4
100	38	N/D	24	16	41	38	10	N/D	13
100.01	50			22			4		

Fig 3A

PIC	IL1/TNF	IL1/IFN	TNF/IFN
23	5.1	8.3	2.7
6.8	13	15	2.3
4.3	6.5	3.2	0.8
4.3	6.1	3.5	1.2
4	4.3	2.8	2.8
10	10	6.6	1.2

Fig 4A

PIC	+ Ab
128	98
101	97
80	88
91	52
114	43
131	73
91	

Fig 5A

PIC	+ 100nM
100	67
100	72
100.01	78
100.001	111
100	39
100	92
100.01	58
100.001	67
100	101
100	72
100.01	
100.001	

Fig 6A

PIC	+ LSF
24.39	38.32
51.56	51.56
61.32	41.81
93.37	104.52
202	299.65
146.34	97.56
174	334.49
313.58	188.15
181.18	41.11
22.29	202.09
132.4	76.65
52.26	48.78
43.9	68.29
52.96	18.11
22.29	13.24
25.78	

Fig 7A

Ctl	PIC	+ c47
128.91	180.05	127.07
128.59	131.42	127.58
122.11	159.08	126.47
		125.3
		120.95
		123.59

Fig 8A

PIC	+ Ab
95	70
70	83
89	63
146	62
82	68
128	98
99	65
91	

Fig 8B

PIC	+ STA-5326
100	130
100	97
100.01	78
100.001	68
100	132
100	65
100.01	61
100.001	62
100	50
100	69
100.01	
100.001	

Fig 8C

PIC	+ LSF
95	61
93	65
163	72
112	43
133	97
78	100
65	133
101	85
163	112
102	44
63	49
59	22
72	57

Fig 8D

PIC	+ c47
82.13	39.74
80.37	74.19
141.3	60.94
97.15	66.24
114.81	105.98
67.12	50.34
87.43	
141.31	
88.32	

Fig 9B

Ctl	PIC	IL1/TNF	IL1/IFN	TNF/IFN
65.396	74.132	70.674	80.082	74.034
73.826	78.098	71.309	76.655	51.864
81.504	78.66	75.078	77.676	52.44
79.215	81.417	68.18	75.18	68.499
68.908	71.767	67.893	80.892	64.038
62.652	80.091	76.681	90.83	64.475
70.858	75.239	67.39	96.092	54.066
79.676	75.605	74.64	81.356	92.195
82.567	67.172	90.409	86.82	72.07
72.201	63.557	89.348	81.923	77.587
69.889	70.089	86.2		67.174
74.971		79.61	79.855	76.523
86.997	72.447	80.197	81.026	88.546
78.595	78.998	90.556	86.674	90.037
79.319	73.795	77.293	93.159	69.128
	88.57	73.58	73.89	52.628
74.162	80.221	76.859	71.733	
72.916	74.997	79.529		70.404
83.361	87.17	70.391	73.465	78.975
69.538	80.17	77.359	76.181	85.459
69.35	84.46	89.72	70.236	77.129
72.117	75.186	68.701	84.78	70.338
71.797	78.945	77.983	81.776	
75.505	74.078	87.405	80.481	71.092
	83.534	71.241	78.883	70.867
69.444	78.646		81.079	77.114
65.789	76.788	77.349	75.983	93.3
76.957	81.44	80.28	71.48	77.424
78.354	78.217	86.086	74.057	79.026
73.362	80.015	89.267	78.246	78.787
75.173	80.986	84.577		71.998
70.261	75.983	88.22	81.917	73.338
83.04	73.338	77.359	76.565	77.197
71.299		80.954	81.376	70.951
66.672	72.279		85.165	76.791
63.389	74.489	64.825	81.98	69.982
	78.521	57.538	79.076	69.546
75.306	77.223	58.763		69.954
74.8	88.471	68.328	74.495	82.318
78.866	77.608		78.637	68.373
82.131	72.179	60.092	83.877	68.128
76.869	77.142	69.088	83.182	66.816
74.185	77.209	61.332	83.574	68.602
79.792		60.957	80.505	73.873
75.139	61.209	60.918	79.676	75.426
	62.304	56.328	77.021	76.09
79.839	64.381	59.1	78.136	73.824
73.131	63.805		72.729	
72.814	78.199	58.44	77.14	74.456
91.662	60.249	62.253	81.415	71.413
77.353	59.913	62.934	75.733	70.482
86.038	65.772	63.913	74.402	77.392
74.52	60.69	58.88		80.39
74.191	59.776		73.834	82.045
78.667		74.274	72.589	71.821
77.685	74.522	73.371	77.153	60.225
80.346	73.339	73.339	73.243	
76.434	72.308	77.243	76.016	68.072
79.968	69.272	73.176	79.504	67.992
	71.032	75.579	73.682	66.93
69.701	74.245	66.357	78.709	71.657
71.689	72.628	69.093	75.174	69.917
83.641	74.17	62.207	69.53	69.619
74.945	70.265	65.519	70.132	71.514
78.416	76.029	67.688	74.911	66.527
74.465	74.241	72.105	70.901	98.842
74.662	73.097	70.076		84.975
75.12	71.275	79.907		70.016
75.286	75.168	53.964		65.595
75.606	72.524	56.726		62.569
74.955	74.146	55.23		61.68
73.023	76.645	59.654		77.422
73.513	72.986	60.264		83.524
75.298	76.211	52.067		74.099
73.488	74.599	52.861		75.976
76.521	73.915	51.218		71.754
				76.762
				73.794
				80.347
				72.297
				68.91
				78.645
				73.485

94.058	99.125	62.678	63.37	95.122
75.133	79.16	70.689	97.526	105.579
81.098	84.56	66.375	97.516	79.855
79.008	95.742	64.143	69.891	84.806
132.488	70.191	63.786	92.799	74.804
103.232	104.258	61.53	92.273	90.742
87.153	72.464	60.027	95.168	79.93
64.822	105.562	75.616	89.679	72.737
61.549	84.585	64.515	77.114	115.42
79.889	77.536		98.063	74.129
95.997	68.608	79.57	92.581	87.777
119.622	84.55	79.861	142.232	69.835
	85.085	78.514	132.928	85.415
69.303	92.236	66.706	95.811	82.307
64.692	117.514	78.784	69.213	75.549
65.859	92.731	119.455	78.059	64.711
79.008	75.71	94.193	69.347	102.37
80.634	68.634	101.332	82.378	75.958
70.696	61.373	109.028	69.78	70.249
108.05	72.014	128.582	140.841	83.744
70.041	71.201	103.779	80.218	
80.662	73.163	74.933	74.92	61.962
68.952	104.203	80.389	86.575	66.196
81.581	82.564	69.241	67.002	61.336
63.315	82.008	70.314		60.828
66.866	75.734	98.78	57.794	58.304
70.238	76.327	120.068	55.617	61.712
63.85	75.848	77.327	66.445	60.314
62.315	76.14	100.981	54.556	73.102
	78.173	95.218	73.83	51.512
107.809	63.825	70.751	95.32	60.681
96.529	62.738	85.816	64.235	
92.454		75.216	74.579	81.316
66.944	63.306	62.911	59.613	66.428
71.504	54.484	79.266	65.946	56.495
72.655	55.714	71.916		64.849
63.899	71.943	62.284	69.683	55.761
66.517	75.308	62.572	69.935	60.467
73.561	73.653	61.7	78.88	
63.998	67.989	63.357	92.228	78.754
58.77	73.103	61.146	112.042	72.08
	82.776	64.021	63.882	87.572
	70.277	86.356	59.395	101.382
	68.059	85.051	64.368	77.512
	75.694	89.327	60.332	111.704
	62.419	81.432	58.289	143.593
	71.252	88.738	72.553	70.068
	67.31	72.247	80.065	75.383
	102.233		84.063	70.363
	88.187	89.08	78.431	91.589
	67.761	91.363	107.428	108.289
		88.774	79.774	99.752
	132.147	82.527	97.18	91.666
	96.702	79.125	68.977	116.05
	95.928	98.782		125.044
	68.929	118.187	62.626	96.486
	110.683	132.493	129.105	92.562
	106.902	87.087	66.465	98.007
	98.988	88.897	60.435	82.259
	81.282	93.505	79.512	71.184
	120.419	170.67	73.534	77.883
	87.361	109.35	87.053	77.666
	110.57	100.362	83.152	87.284
	129.271	138.977	75.345	93.32
	126.182	87.852	61.483	76.647
	144.069	112.562	63.151	119.692
	91.849	114.923		110.711
	83.335	90.103		127.002
	92.186	92.868		100.854
	97.466	95.396		116.16
		88.372		104.21
	68.373	81.695		87.45
	98.904	77.901		103.742
	107.866	82.75		
	68.147	137.556		74.997
	77.842	80.215		87.367
	67.267	84.81		86.185
	68.821	86.138		100.655
	65.139	81.288		74.007
	86.047	82.799		84.511
	80.477	99.134		85.045
	67.758	113.457		91.208
	78.975			109.505
		57.503		76.847
	129.759	61.023		89.767

Fig 1D

Ctl	PIC	IL1 1x	IL1 4x	TNF 1x	TNF 4x	IFN 1x	IFN 4x
7.818	0	0	0	0	2.361	1.56	0.429
4.629	0	22.156	10.639	0	13.584	0	0
0	0	27.377	2.348	0	5.534	0	0
5.935	0	15.24	5.988	2.595	4.561	0	0
7.134	1.551	17.48	4.698	10.963	3.143	0	7.815
14.179	0.268	6.398	0.795	4.528	0	28.119	32.729
15.811	5.02	0	0	0	0	7.54	18.087
0.228	24.857	3.687	3.581	0	19.622	0	10.81
0	17.98	8.178	0.337	5.148	0	0	6.902
0	10.606	0	0	0	8.824	2.242	3.51
0.131	14.862	16.142	11.79	16.38	7.013	27.651	8.232
1.298	2.151	2.206	7.525	23.491	16.699	14.047	0.509
1.085	18.411	7.329		36.462	0	32.25	0
0	16.238	12.764		30.381	6.388	0	1.142
0	6.484	0	0	16.678	16.937	0	5.729
2.887	3.006	0	3.538	5.474	13.12	0	3.08
0.421	3.472	2.385	0	0	10.497	10.372	0
8.967	0	0	0.622	0	8.566	0	3.072
4.282	0	30.352	11.56	0	6.744	16.414	0
0	0	26.19	12.956	0	3.423	0	0
9.832	2.668	24.251	8.072	0	5.725	0	0
0	0	27.652	4.953	0	7.64	10.03	0
1.636	0.974	6.757		0	0.138	11.592	0
1.713	10.756	7.875		0	0	10.028	0
8.093	0	6.197	10.588	0	0	2.962	0
3.563	47.899	3.667	1.03	0	0	1.243	0
0	42.769	0	7.696	3.059	1.541	1.083	0.292
12.44	34.112	4.368	7.926	0	0	0	0
	31.05	10.937	4.2	14.331	0	0	0
	38.072	3.096	2.524	9.7	0	0	0
5.312	30.539	0.727	3.757	0	0	0	0
5.165	43.268	6.401	7.883	0	5.111	9.207	1.622
11.272	31.19	0	9.456	13.339	0.513	1.863	4.88
15.153	0	0.327	6.835	0	0	0	0
23.824	1.293	4.114	7.235	10.129	0	0	0
0	7.092	4.042	10.708	0	7.496	5.046	0
9.261	38.711	0.597	0	20.33	3.028	0	0
45.01	16.67	4.22	0.1	17.956	0	0	0.501
23.471		2.802	0	0	0.458	0	0
0		2.7	4.092	3.127	0	11.802	0
0	10.936	0	0	0	1.459	16.892	0
1.038	16.584	0	4.283	0	0.812	0	4.502
0	9.49	0	5.949	0.95	0	0	15.776
0	17.652		8.999	0	0	11.256	0
0.972	18.051		6.463	0.999	0	7.208	0
3.182	15.082		8.65	7.995	2.929	9.594	0
0	11.166		1.751	0.162	0	0	0
3.514	18.236		4.295	4.633	7.239	6.728	0.578
	5.765		4.834	8.502	0	0	5.51
	15.487		2.347	9.203	0	0	5.154
4.741	22.555		1.961	3.237	1.38		4.875
1.54	28.837		0.521	0.873	0		0
0	19.162		12.326	2.187	0		4.893
0	16.564		0		0		0
1.896	15.987		8.219		0		0
4.412	35.493		0.458		0		0
4.525	4.746		0		0		0
0	21.665		2.219		0		0
0	25.496		3.253		7.828		0
0	19.948		0		3.249		0.588
0	3.259		0.76		0		2.698
5.087	19.354		0		2.577		5.128
12.322	27.774				2.401		0
0	24.885				0		6.465
0	31.482				0		8.285
0	21.098				1.897		7.183
0	29.106				2.135		0
0	13.088				1.84		0
0	14.206						1.233
	11.983						8.873
	11.577						
26.769	4.886						
31.041	11.267						
39.331	5.182						
18.084							
26.622							
30.136	0						
29.791	0						
33.342	12.081						
2.1	16.685						
0	18.424						
15.543	30.756						
6.272	23.01						
23.179	24.128						
22.639	23.751						
6.445	34.487						
14.005	23.26						
5.093	36.973						
8.136	29.034						
13.453	17.091						
23.9	31.923						
33.333	28.07						
0.762	21.262						
0	17.567						
10.804	15.368						
9.029	7.256						
0	14.436						
3.018	13.135						
	13.149						
0.74	10.666						
11.586	15.036						
0	9.925						
14.303	10.689						
0.072	13.106						
22.021	0						
1.771	10.127						
1.379	22.581						
3.138	5.248						
3.9	10.771						
5.294	26.657						
1.388	25.07						
4.86	2.629						
7.975	24.275						
2.333	53.923						
8.868	14.612						
0	58.118						
6.878	11.501						
6.154	15.953						
10.38	60.72						
0	14.22						
3.238	21.309						
0	30.913						
17.457	27.133						
9.031	18.409						
0	22.174						
3.703	11.087						
5.814	28.004						
3.308	9.019						
0.88	22.046						
0	12.603						
	21.48						
	4.097						
0	4.355						
0	4.624						
0	38.86						
3.103	39.738						
0	39.772						
0.459	36.338						
0	21.102						
	8.545						
	11.12						
5.081	2.537						
0	31.535						
1.326	23.156						

Fig 2B

PIC	IL1/TNF	IL1/IFN	TNF/IFN
100	90	48	20
100	52	42	6
100.01	68	58	25
100	86	59	45
100.0001	59	55	42
100	100	63	56
100	93	87	87

Fig 3B

PIC	IL1/TNF	IL1/IFN	TNF/IFN
2.9	2.8	4.2	2.3
2.5	7	3.7	1.6
4	1.4	1.4	1.4
	0.8	0.3	0.4
	1.4	1.2	1.6

Fig 4B

PIC	+ Ab
581.31	561.17
645.66	521.8
650.68	494.31
615.04	449.48
542.78	588.48
628.37	413.81
516.6	440.63
467.89	451.42
475.5	380.77
431.17	499.97
520.02	401.58
492.49	527.33
501.65	515.92
496.56	547.8
598.93	479.8
527.38	442.14
507.49	471.2
511.75	
554.84	
521.05	

Fig 5B

PIC	+ 100nM
100	48
100	62
100.01	78
100.001	83
100	47
100	97
100.01	57
100.001	41
100	59
100	76
100.01	
100.001	

Fig 5C

PIC	+ 100nM
100	137
100	68
100.01	113
100.001	81
100	111
100	186
100.01	67
100.001	51

Fig 5D

PIC	+ 1nM	+ 100nM
725.2	644.39	497.91
717.5	647.88	522.3
597.92	581.65	498.18
674	650.13	520.32
586.45	540.02	542.16
800.4	638.27	362.6
625.25	553.23	459.54
789	566.41	406.78

Fig 6B

PIC	+ LSF
55.57	59.85
158.17	89.77
38.47	42.75
89.77	102.6
153.9	260.78
183.82	98.32
158.17	188.1
115.42	106.87
85.5	136.8
111.15	76.95
94.05	68.4
102.6	72.67
51.3	76.95
89.77	38.74
55.57	34.2
38.47	21.37
38.47	

Fig 6C

Ctl	PIC	+ LSF	+ c47
469.8	467.5	386.06	470.13
515.71	442.73	394.1	363.7
406.94	536.62	400.96	349.54
474.95	429.63	362.22	345.95
438.69	449.69	435.4	444.56
388.25	474.76	430.97	418.55
381.75	456.11	440.23	386.06
391.07	430.4	439.78	394.1
365.51	460.73	406.99	400.96
340.78	436.9		362.22
349.45	436.36		435.4
395.57	439.67		
352.11			
375.19			
392.62			
405.47			

Fig 6E

Ctl	PIC	+ LSF	+ c47
0.11	0.45	0.2	0.12
0.11	0.96	0.38	0.35
0.11	0.58	0.2	0.19
0.11	0.95	0.49	0.58
0.1	1.11	0.6	0.64
0.11	1.47	0.4	0.7
0.13	1.49	0.62	

Fig 7C

Ctl	PIC	+ c47
0.67	1.13	0.32
0.44	1.43	0.27
0.51	0.82	0.28
0.53	0.89	0.29
0.5	0.86	0.45
0.66	0.95	0.37
0.81	0.94	0.66
0.68	0.98	0.68
0.81	0.9	0.81
0.88	1.78	0.78
0.62	1.05	0.59
0.5	1.04	0.45
0.61	0.72	
	0.66	
	1.12	
	0.72	

5.049	33.539
14.812	21.27
0.63	19.096
8.052	35.461
0	7.334
7.164	5.653
0	5.051
7.002	9.183
0	5.287
0	9.719
0	5.801
0	6.217
0	2.765
0.727	2.76
5.785	2.069
1.222	8.361
0	3.387
0	2.223
0	5.046
	3.087
	8.083
8.439	4.765
14.572	4.629
18.057	12.935
3.475	10.405
27.687	7.729
35.085	6.792
6.757	3.806
16.03	6.431
36.732	0.73
57.903	6.09
50.829	5.95
13.11	1.784
10.656	6.997
15.329	20.242
0	22.454
0.618	0.6
	10.983
	25.069
10.869	18.862
9.826	12.225
0.888	19.144
10.085	1.021
3.362	13.907
6.908	12.251
5.391	11.966
0.946	0
17.483	0.459
0	2.379
4.031	11.568
9.66	0
14.735	5.823
22.563	12.676
22.354	2.604
22.084	6.647
	20.135
	12.381
0	15.531
3.307	9.736
7.798	0
8.694	35.425
0	0
0	5.109
0	1.528
7.974	2.129
4.534	5.164
4.751	0
13.601	1.875
10.543	4.169
0	7.159
	2.43
	2.657
0	6.791
17.439	6.819
11.631	7.41
2.711	4.002
2.92	1.761
0	3.595
0	2.567
0	4.666
16.394	5.609
10.966	9.774
	0
	7.416
0	11.774
0	7.227
0	7.876
0	3.182
3.465	6.508
1.14	9.56
0	5.776
5.892	4.365
0	12.148
7.848	13.696
	4.238
	6.438
0	7.057
8.374	0.785
19.352	8.96
19.151	8.75
19.611	22.188
14.955	11.588
5.007	11.804
5.388	
7.59	
22.108	
23.153	
0	
3.557	55.527
2.068	20.168
13.434	33.826
14.255	61.451
21.569	75.777
21.383	43.267
	20.54
	29.201
4.754	72.819
6.653	44.982
4.327	68.113
5.245	36.036
8.982	75.226
0	45.873
0	74.476
8.509	29.096
9.906	37.062
3.195	78.254
14.327	88.81
5.603	24.311
7.06	70.965
	41.847
	51.159
	50.174
	62.979
	64.986
	63.701
	72.171
	80.314
	58.488
	32.067
	39.029
	4.722
	0
	0.966
	1.148
	7.402
	5.25
	17.863
	0.529
	0
	2.785
	0
	4.925
	0
	8.095
	5.642
	9.718
	21.794
	22.224
	32.456
	29.191
	8.432
	17.41
	14.838
	11.856
	2.567
	7.722
	0.456
	6.729
	1.471

127.914	63.976	74.488
118.567	56.226	99.374
105.051	60.479	131.987
85.232	97.891	100.322
74.068	109.903	72.06
79.965	126.847	75.799
83.968	119.577	67.209
96.576	158.016	121.075
97.848	120.818	88.028
81.986	72.895	117.945
101.709	82.879	93.795
72.895	65.494	79.055
74.572	85.687	73.551
	125.239	60.03
	74.055	74.28
	78.646	60.637
	70.164	
	55.758	
	59.787	
	55.75	
	59.588	
	54.077	
	56.921	
	66.783	
	95.496	
	78.071	
	69.562	
	57.27	
	72.464	
	80.723	
	63.414	

Fig 9C

Control	PIC	IL1/TNF	IL1/IFN	TNF/IFN	STAT4ko Ct	STAT4ko PI	AT4ko IL1/T	AT4ko IL1/I	AT4ko TNF/IFN
0.3184104	1.249337	0.8900363	1.282636	0.5607601	0.853949	0.9680248	0.9228698	1.045721	0.966745
0.3478106	1.555374	0.8376308	0.5637235	0.4994383	0.9640289	1.019813	0.9311616	1.00097	0.6772465
0.3666048	1.351704		1.001138	0.6768529	1.064289	1.027152	0.9803777	1.014303	0.6847679
0.365747	1.216063	0.5565229	1.463508	0.4286544	1.034399	1.063153	0.8903028	0.9817097	0.8944683
0.3249612	2.850929	1.030408	1.487215	0.3477066	0.8998091	0.9371423	0.8865551	1.056298	0.8362161
0.3375426	1.173406	1.906096	1.35459	0.4542073	0.8181175	1.045838	1.00131	1.186069	0.8419224
	1.007741		0.5319579	0.4090804	0.9252725	0.98248	0.8799869	1.254781	0.7060004
	0.8639635		1.297141	0.4532715	1.040419	0.9872594	0.9746583	1.062357	1.203894
	0.8220599				1.07817	0.8771402	1.180572	1.133706	0.9410989
	1.023442				0.9428095	0.8299351	1.166717	1.069761	1.013141
					0.9126191	0.9152307	1.12561		0.8771663
					0.9789805		1.039557	1.042756	0.9992467
					1.136017	0.9460218	1.047222	1.058047	1.156245
1.580952	1.520566	1.638323	1.257525	1.631148	1.026303	1.031566	1.182491	1.1318	1.175714
1.319393	1.430573	1.532628	1.220145	1.407723	1.035757	0.9636241	1.009301	1.216482	0.9026819
1.683502	1.346011	1.136259	1.555503	1.167219		1.156558	0.9608167	0.9648647	0.6872228
1.546847	1.232492	1.499771	1.571542	1.077121	0.9684165	1.047536	1.003634	0.9366983	
		1.156743	1.204236	0.781378	0.9521461	0.97932	1.038499		0.919344
		1.113826	0.8821079		1.088538	1.138276	0.9191743	0.9593149	1.031265
					0.9080358	1.04687	1.010163	0.9947808	1.115934
					0.9055808	1.102889	1.171575	0.9171503	1.00716
					0.9417126	0.9817879	0.897106	1.107068	0.9184822
1.407463	1.600527	1.932974	1.616201	1.65171	0.937534	1.030873	1.018312	1.067841	
1.610067	1.681084	1.906875	1.575104	1.365923	0.9859535	0.9673196	1.141345	1.050931	0.9283281
1.335042	1.645004	1.560287	1.73375	1.583136		1.090797	0.9302737	1.030064	0.9253899
1.455657	2.152085	1.597303	1.604478	1.67482	0.9068083	1.026969		1.05874	1.006964
	1.560729	1.575026	1.467433	1.795929	0.8590809	1.002707	1.010033	0.9921953	1.218323
		1.457581	1.569047		1.004914	1.063454	1.048306	0.9333946	1.011012
		1.38451	2.319491		1.023156	1.021367	1.124122	0.9670454	1.031931
			1.638973		0.95797	1.044846	1.165659	1.021746	1.02881
					0.9816182	1.057525	1.104417		0.9401587
					0.9174768	0.9921953	1.151988	1.069682	0.9576566
					1.084347	0.9576566	1.010163	0.9997952	1.008048
					0.9310311		1.057107	1.062618	0.9264868
					0.8706111	0.943828		1.112095	1.002746
					0.8277413	0.9726865	0.8464928	1.070505	0.9138336
						1.025337	0.7513382	1.032584	0.9081401
					0.983355	1.008387	0.7673345		0.9134679
					0.9767476	1.155265	0.8922354	0.9727648	1.074919
					1.029842	1.013415		1.026852	0.892823
					1.072477	0.9425222	0.7846887	1.095276	0.8896238
					1.003765	1.00733	0.9021595	1.086201	0.8724915
					0.9687168	1.008205	0.8008808	1.091319	0.8958133
					1.041934		0.795984	1.051244	0.9646427
					0.9811742	0.7992746	0.7954747	1.040419	0.984922
						0.8135732	0.7355379	1.00575	0.9935925
					1.042547	0.8406949	0.771735	1.020309	0.9640028
					0.9549535	0.8331735		0.9497041	
					0.9508142	1.021132	0.7631167	1.007304	0.9722556
					1.196934	0.7867388	0.8129073	1.063127	0.9325197
					1.010085	0.7823513	0.8217999	0.9889308	0.9203626
					1.123495	0.8588589	0.8345838	0.9715504	1.010594
					0.9730912	0.7924975	0.7688622		1.049742
					0.9687952	0.7805623		0.9641334	1.071354
					1.027243		0.969879	0.947876	0.9378474
					1.01442	0.9731174	0.9580875	1.007473	0.7864254
					1.049168	0.9576696	0.9576696	0.956416	
					0.9980845	0.9442067	1.008649	0.9926262	0.8888925
					1.044232	0.9045623	0.9555412	1.038173	0.8878478
						0.9275445	0.9869199	0.9621485	0.8739801
					0.9101641	0.9695003	0.8664979	1.027792	0.9357058
					0.9361238	0.9483853	0.9022249	0.9816313	0.9129847
					1.092194	0.9685209	0.8123066	0.9079312	0.9090934
					0.978641	0.917529	0.8555551	0.9157923	0.9338385
					1.023966	0.992796	0.8838782	0.978197	0.8687177
					0.972373	0.969448	0.941556	0.9258339	1.290691
					0.9749455	0.9545096	0.9150609		1.109614
					0.9809262	0.9307177	1.043435		0.9142775
					0.9830939	0.981553	0.7046685		0.8565475
					0.9872724	0.9470273	0.7407351		0.8170336
					0.9787716	0.9682076	0.7212001		0.805425
					0.9535433	1.00084	0.7789692		1.010986
					0.9599417	0.9530601	0.7869347		1.090667
					0.9832504	0.9951726	0.6798972		0.9675938
					0.9596153	0.9741229	0.6902654		0.9921039
					0.9992206	0.9651911	0.6688109		0.9369725
									1.002368
									0.9636111
									1.049181
									0.944063
									0.8998353
									1.026956
									0.9595761
					1.228221	1.294386	0.818457	0.8274932	1.242115
					0.981096	1.033681	0.9230657	1.273506	1.378664
					1.058988	1.104195	0.8667329	1.273376	1.042756
					1.031696	1.250211	0.8375871	0.9126452	1.107407
					1.730045	0.9165627	0.8329254	1.211781	0.9767998
					1.348016	1.361414	0.8034663	1.204912	1.18492
					1.138054	0.9462438	0.7838399	1.242715	1.043736
					0.8464536	1.378441	0.9874029	1.171039	0.9498087
					0.8037144	1.104521	0.8424448	1.006964	1.507168
					1.0432	1.012475		1.280519	0.9679855
					1.253541	0.8958917	1.039035	1.208934	1.146203
					1.562039	1.104064	1.042835	1.857283	0.911914
						1.11105	1.025245	1.73579	1.11536
					0.9049671	1.204429	0.8710551	1.251112	1.074775
					0.8447561	1.534512	1.028771	0.9037918	0.9865282
					0.8599949	1.210893	1.559858	1.019304	0.8450041
					1.031696	0.9886304	1.229984	0.9055416	1.33676
					1.052929	0.8962312	1.323206	1.075702	0.9918689
					0.923157	0.8014162	1.423701	0.9111958	0.9173201
					1.41093	0.9403676	1.679039	1.839119	1.093539
					0.9146039	0.9297513	1.355159	1.047496	
					1.053294	0.9553714	0.9784843	0.9783145	0.8091074
					0.9003837	1.360696	1.049729	1.130507	0.8643954
					1.065295	1.078131	0.9041574	0.8749203	0.8009329
					0.826775	1.070871	0.9181688		0.7942995
					0.8731444	0.9889439	1.289881	0.7546811	0.7613408
					0.9171764	0.9966874	1.567863	0.7262536	0.8058429
					0.8337611	0.9904324	1.009745	0.8676469	0.7875876
					0.8137169	0.9942454	1.318622	0.7123989	0.9545748
						1.020793	1.243368	0.9640812	0.67265
					1.407783	0.8334346	0.9238752	1.2447	0.7923799
					1.260487	0.8192405	1.120596	0.8387885	
					1.207276		0.9821798	0.9738618	1.061834
					0.874163	0.8266575	0.8214995	0.7784339	0.867425
					0.933708	0.7114587	1.035065	0.861131	0.7377186
					0.9487379	0.7275202	0.9390879		0.8468062
					0.834401	0.9394405	0.8133121	0.9099291	0.728134
					0.8685871	0.983381	0.8170728	0.9132197	0.7895855
					0.9605685	0.9617699	0.8056862	1.030025	
					0.8356937	0.8878087	0.8273234	1.204324	1.028379
					0.7674258	0.9545879	0.798452	1.463058	0.9412295
						1.080899	0.8359941	0.834179	1.143526
						0.9176857	1.127647	0.7755872	1.323859
						0.8887227	1.110606	0.8405252	1.012161
						0.9884215	1.166443	0.7878227	1.458645
						0.8150749	1.063349	0.7611449	1.875055
						0.9304173	1.158752	0.947406	0.9149565
						0.8789422	0.9434102	1.045499	0.9843605
						1.334971		1.097705	0.9188086
						1.151557	1.163218	1.024162	1.19598

5.204
11.114
4.906
18.652
26.591
5.595
12.971
4.965
11.554
9.664
18.547
23.524
14.244
3.801
6.768
12.302
2.294
23.045
12.128
1.389
3.675
12.926
10.578
0.877
1.322
5.089
9.002
3.352
2.826
15.831
8.7
33.314
0
0
15.395
0.829
5.51
4.967
5.285
7.724
8.248
5.449
9.365
0
0
0.807
4.4
37.133
19.479
16.4
36.091
15.735
13.445
21.485
36.559
45.04
28.947
13.169
8.685
5.993
1.406
9.741
7.033
2.2
25.048
18.741
45.122
45.693
50.071
38.267
40.577
0
0
0
15.712
11.312
50.825
80.862
88.235
74.66
75.072
28.091
64.549
37.322
40.423
5.291
25.829
17.273
11.597
22.398
13.045
41.393
23.436
45.335
33.415
20.163
48.585
6.101
8.287
0.246
0
3.98
0.941
0
17.43
12.21
6.696
12.781
27.815
24.342
51.908
49.324
48.855
39.697
2.728
0.697
7.603
0.791
0
1.682
5.937
11.723
6.61
4.638
22.052
27.042
56.537
65.77
30.467
74.631
14.472
37.285
45.087
21.77
36.512
11.908
7.708
1.59
8.413
1.715
3.18
7.351
6.415
4.295
12.657
10.564
35.979
52.221
35.765
42.849
29.984
50.92
50.986
57.039
32.38

0.8848314	1.193029	1.402808	1.414051
	1.159222	1.041699	1.302574
1.725592	1.077648	1.268988	1.196986
1.262747	1.033224	0.90071	1.515395
1.25264	1.289907		1.63284
0.9000833	1.5433	0.817778	1.259926
1.445312	1.73011	1.685869	1.208686
1.395939	1.137193	0.8679081	1.279787
1.292597	1.160828	0.7891676	1.074148
1.06139	1.221	1.038277	0.9295294
1.572446	2.22863	0.9602159	1.017006
1.140771	1.427906	1.136749	1.014172
1.443837	1.310539	1.085809	1.139765
1.688036	1.814779	0.9838642	1.218584
1.6477	1.147182	0.8028526	1.000866
1.881271	1.469848	0.8246335	1.562953
1.199376	1.500679		1.445678
1.088199	1.176576		1.658408
1.203776	1.212682		1.316964
1.272723	1.245693		1.516832
	1.153972		1.360787
0.892823	1.066783		1.141933
1.291501	1.017241		1.354676
1.408527	1.08056		
0.8898719	1.796223		0.97932
1.01647	1.047457		1.140849
0.8783807	1.107459		1.125414
0.898673	1.124801		1.314365
0.850593	1.061469		0.9663925
1.123612	1.0812		1.103555
1.050879	1.294504		1.110528
0.8847923	1.481535		1.191005
1.031265			1.42993
	0.7508812		1.003478
1.694409	0.7968458		1.172188
1.670317	0.8354065		0.9726734
1.548262	0.734206		1.297638
1.371769	0.7897422		1.723502
1.11297	1.278273		1.310017
0.967189	1.435127		0.9409683
1.044193	1.656384		0.9897926
1.096464	1.561451		0.8776233
1.261101	2.063392		1.581012
1.277711	1.577656		1.14948
1.070583	0.9518718		1.54014
1.328129	1.082244		1.224787
0.9518718	0.8552287		1.03231
0.9737703	1.118911		0.960438
	1.635386		0.783879
	0.9670193		0.9699573
	1.026969		0.7918054
	0.9162101		
	0.7280948		
	0.7807059		
	0.7279903		
	0.7781074		
	0.7061441		
	0.7432814		
	0.8720605		
	1.246998		
	1.019461		
	0.908349		
	0.7478387		
	0.9462438		
	1.054091		
	0.8280678		

53.986
45.234
69.05
32.499
93.672
51.096
32.694
46.565
42.065
12.209
4.681
16.329
13.977
8.125
13.6
8.757
0
5.13
0
0
17.041
5.742
37.864
43.813
42.84
31.701
45.456
44.946
52.577
30.38
47.682
44.521
40.771
32.976
8.997
15.577
5.859
3.925
7.668
13.749
8.208
44.845
46.672
67.583
55.639
27.172
73.19
72.758
38.355
46.067
33.582
64.244
32.996
23.222
30.307
26.188
29.483
36.305
25.82
24.65
0.515
0.221
4.535
7.544
3.419
19.217
10.764
5.66
14.749
11.387
17.131
16.184
